# Supplementary material for: Apomixis and genetic background affect distinct traits in Hieracium pilosella L. grown under competition
Source: BMC Biol. 2021 Aug 28;19:177. doi: 10.1186/s12915-021-01117-x (PMC8403437; doi:10.1186/s12915-021-01117-x)
Supplement: Supplementary file 2 — Additional file 2: SFigure 1. Experimental design. [file 12915_2021_1117_MOESM2_ESM.pdf]

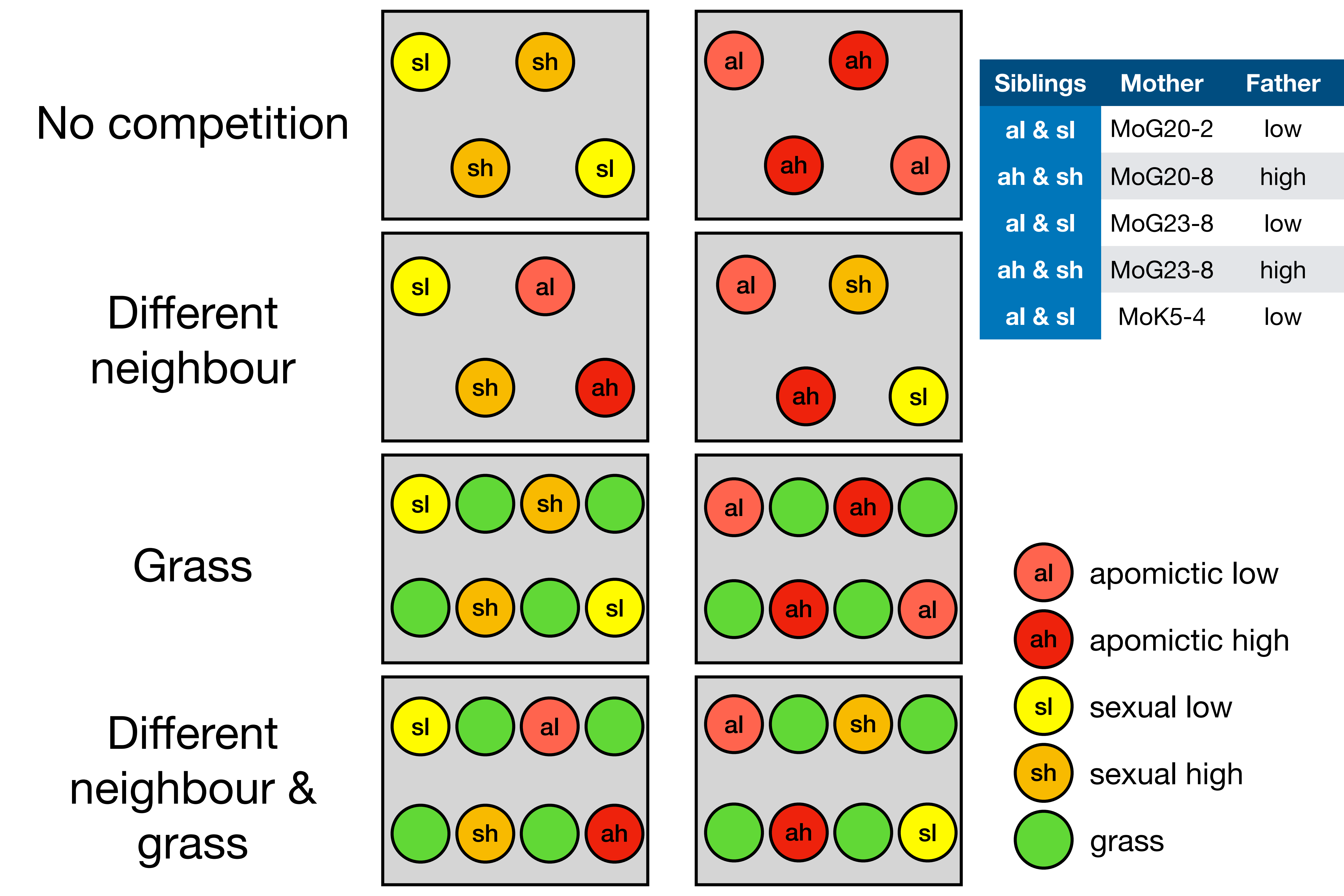

Siblings	Mother	Father
al & sl	MoG20-2	low
ah & sh	MoG20-8	high
al & sl	MoG23-8	low
ah & sh	MoG23-8	high
al & sl	MoK5-4	low
